# Antiepileptic drug utilization in Bangladesh: experience from Dhaka Medical College Hospital

**DOI:** 10.1186/1756-0500-6-473

**Published:** 2013-11-18

**Authors:** Mansur Habib, Sharif Uddin Khan, Md Azhahul Hoque, Md Badrul Alam Mondal, ATM Hasibul Hasan, Rajib Nayan Chowdhury, Badrul Haque, Kazi Mohibur Rahman, Ahmed Hossain Chowdhury, Swapon Kumar Ghose, Quazi Deen Mohammad

**Affiliations:** 1 Department of Neurology, Dhaka Medical College Hospital, Dhaka, Bangladesh; 2 Department of Medicine, Dhaka Medical College Hospital, Dhaka, Bangladesh

**Keywords:** Anti epileptic drug (AED), Effective, Treatment retention rate

## Abstract

**Background:**

Epilepsy is a common health problem which carries a huge medical social psychological and economic impact for a developing country. The aim of this hospital-based study was to get an insight into the effectiveness and tolerability of low cost antiepileptic drugs (AEDs) in Bangladeshi people with epilepsy.

**Methods:**

This retrospective chart review was done from hospital records in weekly Epilepsy outdoor clinic of Department of Neurology, Dhaka Medical College Hospital (DMCH) from October 1998 to February 2013. A total of 854 epilepsy patients met the eligibility criteria (had a complete record of two years of follow up data) from hospital database. A checklist was used to take demographics (age and gender), epilepsy treatment and adverse event related data. At least two years of follow up data were considered for analysis.

**Results:**

Out of 854 patients selected, majority of the patients attending outdoor clinic were >11-30 years age group (55.2%) with a mean age of 20.3 ± 9 years and with a male (53%) predominance. Focal epilepsy were more common (53%), among whom secondary generalized epilepsy was the most frequent diagnosis (67%) followed by complex partial seizure (21%). Among those with Idiopathic Generalized Epilepsy (46%), generalized tonic clonic seizure was encountered in 74% and absence seizure was observed in 13%. The number of patients on monotherapy and dual AED therapy were 67% and 24% respectively and polytherapy (i.e. >3 AEDs) was used only in 9%. CBZ (67%) was the most frequently prescribed AED, followed by VPA (43%), PHB (17%), and PHT (8%). CBZ was prescribed in 37% patients as monotherapy followed by VPA in 21% and PHB in 8% patients. Newer generation drugs eg lemotrigine and topiramate were used only as add on therapy in combination with CBZ and VPA in only 2% patients. The treatment retention rates over the follow up period for the AEDs in monotherapy varied between 86 and 91% and were highest for CBZ, followed by VPA. Most of the combination regimens had a treatment retention rate of 100%. The effectiveness of AED in terms of reduction of seizure frequency was highest for PHT (100%) and PHB (98%) followed by CBZ (96%) and VPA (95%). PHB and PHT were the cheapest of all AEDs (42 I$ and 56 I$/ year respectively). The costs of VPA and CBZ were two times and LTG and TOP were six to eight times higher. Adverse drug reaction (ADR) were observed among 140 (24.5%) of those with monotherapy. PHT (64%) was the most common drug to cause ADR, CBZ was at the bottom of the list to cause adverse effect (11.6%). VPA and PHB caused weight gain commonly. Adjustment of drug dose or withdrawal due to ADRs was necessary in 39% with PHT and 26% with PHB.

**Conclusion:**

Though PHT and PHB are cheapest and efficacious among all, CBZ and VPA are less costly, effective and well tolerated drug for seizure control in context of Bangladesh.

## Background

Epilepsy is a common and chronic neurologic disorder worldwide. The risk of having epilepsy at some point in average life span of any individual varies between 2%-5% [1]. Some hospital and community based studies from South East Asian countries (SEAR) have reported the incidence of epilepsy from 2–10 per thousand population [1]. WHO estimates that there are at least 1.5-2.0 million people with epilepsy in Bangladesh [1]. The incidence and prevalence of epilepsy being higher in poor areas (49 to 225 per 100,000 people per year) [2], poses a huge social and economic burden to the poor. About half of the total epilepsy population lives in Asia [3]. Though most of the AEDs are cheap, the volume of prescription and duration of treatment often makes the expenditure high enough for the poor. As the definition implies, epilepsy is a disorder of uncertainty and unpredictability. A seizure event can occur anytime, anywhere without warning; causing embarrassment, injury or even death of the person [4]. This may have a disastrous effect on employment and independence [5]. About 50% of the patients become seizure free with the first drug tried and can lead a normal life [6]. Seizure can be controlled with monotherapy in majority of them [7].

This usually requires careful and rigid adherence to drug regimens, which involve taking tablets regularly, two or three times each day for many years, sometimes for a lifetime. Physicians often judge the effectiveness of treatment by gross clinical response. There are wide variations in each person’s response to treatment, and the plasma concentration of AEDs provides little information about a person’s likelihood of reduction of seizures or side-effects. The available data on effectiveness of drugs are mostly from the western studies [8]. But the drug response and effectiveness may vary depending on the genetic, environmental, pharmacodynamic and pharmacokinetic interaction in different racial groups. Moreover, the cost of AEDs often poses a treatment gap in management of epilepsy, especially in least developed country like Bangladesh. So our aim was to find out the effectiveness and side effect profile of AEDs used in epilepsy clinic of Dhaka medical college, especially the low cost ones.

## Methods

### Sample selection

This is a retrospective chart review, carried out from hospital records in weekly Epilepsy outdoor clinic of Department of Neurology, DMCH from October 1998 to February 2013. During this period a total of 3284 patients with seizure disorder attended the epilepsy clinic of DMCH. Among them, 854 epilepsy patients met our inclusion criteria from hospital database. Patients aged 5 yrs and above with seizure disorders who were in follow up for at least two years from the last AED added and had a complete set of desired information (patients identity, age, sex, occupation, comprehensive clinical history of seizure including duration, frequency, types etc., any associated disorders, examination findings, investigation reports and the drug usage profile with any adverse event) in record files were included in the study.

A questionnaire (Summery of information is listed in next section) was used to take data regarding demographics (age and gender), type and epileptic seizures, age of onset of seizures, frequency of seizure at onset (number of seizure per six months) and at the end of follow up, duration of disease and therapy, duration of follow-up at DMCH, clinical or biochemical evidence of any adverse drug reaction. The epilepsy clinic used simple 1981 international league against epilepsy (ILAE) classification system to label the seizure events in record file (Commission on Classification and Terminology of the International League Against Epilepsy, 1981). So we grouped seizure events simply as partial (complex partial, simple partial, secondary generalized), generalized (generalized tonic-clonic, absence, myoclonic, clonic, tonic, atonic) and unclassified. Those not following any particular type was labeled unclassified.

### List of collected information

I. **Socio-demographic variables:**

1. Age

2. Sex

3. Social status

4. Marital Status

II. **Variables related to Seizure:**

1. Age of onset of seizure

2. Duration of disease

3. Seizure type

4. Classification of epilepsy

5. Duration of Previous Therapy

6. Duration of Follow Up

7. Seizure frequency at first visit

8. Seizure frequency at the end of follow up

9. Percentage reduction of seizure frequency

III. ** Drug related information:**

1. Number of AED used

2. AED dose at initial visit and at every follow up

3. AED dose range

4. Change in seizure frequency

IV. **Adverse drug reaction:**

1. Change in blood picture

2. Skin reaction

3. Change in body weight

4. Liver

5. GIT

6. CNS

7. Eye

A total of 2430 patents were excluded from the study. We had to exclude a large number of patients who did not return to follow up or were in follow up for less than two years. Though we excluded all the patients diagnosed as pseudo-seizure and those with structural brain lesion (tumor, heamatoma, cyst, tuberculoma or any other space occupying lesion), epilepsy patients with comorbid psychiatric illness like anxiety, depression were included in this study. We also excluded all the patients with incomplete hospital record. There was no difference in demography, seizure types or drug utilization pattern between the included or excluded patients.

During the record keeping every patient was examined initially by post graduate trainees and later evaluated and treated by consultant neurologists. Diagnosis was made on clinical ground. EEG and other investigations were done only in required cases. After proper recording EEG were analyzed and reported by two Neurologist of Dept. of Neurology of DMCH. EEG showing focal epileptiform activity was labeled as LRE (Localization Related Epilepsy) and generalized epileptic activity was labeled as GE (Generalized Epilepsy).

### Operational definitions

**Epilepsy**: Tendency to have recurrent seizure.

**Low Cost AED**: Any AED costing 3 taka or less per tablet in local market (1 US $ is equivalent to approximately 82 taka).

**Effectiveness**: It is measured as treatment retention rate at the end of follow up and percentage in reduction of seizure frequency from the time of drug initiation.

**Treatment retention rate**: It is the percentage of patients who were maintained on the initial anti epileptic drug at the end of follow up period.

**Seizure free at the end:** It was evaluated as percentage of patients who are seizure free at the end of follow up period.

**Reduction in seizure frequency:** The frequency of seizure was calculated as the number of seizure events per six month before initial visit and at every follow up visit. The difference of seizure frequency was calculated by subtracting number of events per six month from the previous value. In epilepsy clinic patients are asked to maintain a diary of seizure events with date, time and duration which are kept written in patient record form.

### Antiepileptic drug with effectiveness and tolerability profile

Drug related data included the AED regimen (name, number, dose range) at initial visit and at the end of follow up along with any adverse drug reaction. The effectiveness of antiepileptic was measured by treatment retention rate (the percentage of patients which were maintained on the same AED therapy since the time from their first visit or start of therapy at DMCH (de novo patients) and the number of patients who were seizure free on a particular AED regimen at the end of the follow-up period (at least 2 years after the last drug added). Seizure frequency was recorded as number of seizure events per six months both at the time of first visit and the subsequent follow up. Treatments were provided by experienced neurologists (Professor of neurology to Assistant professor) in the clinic. They also recorded any adverse drug reaction either reported by the patient or found through routine examination and investigation during follow up. Tolerability was measured by extracting data from the record files on the known adverse reactions (any cytopenia in blood, skin reactions, mouth, gastro-intestinal, liver, central nervous system problems, change in body weight etc.) of these drugs. At least 5% gain of weight during follow up was considered to be related to AED usage.

### Cost of antiepileptic drugs

The cost of AEDs were calculated as average price of standard dose per tablet (e.g. CBZ 200 mg tab) in local market from the average of at least 5 top pharmaceutical companies. We collected the price of standard doses form of tablets from the local market. All the drug price of five commonly prescribed pharmaceutical companies were summated and subdivided by five to get the arithmetic mean of the individual drugs. We multiplied the unit price with average maintenance dose of the drug. The rough average annual cost was also calculated. The price of the drug is paid by the patients themselves except for the PHB and PHT which are provided free of cost by the government and available at medical college hospitals and also at local government hospitals. Though PHB is under tight regulation in many countries, it is still readily available in Bangladesh. To adjust the price in regional context the unit price (cost per tablet) was also calculated in International Dollars (I$). An International Dollar has the same purchasing power as the U.S. Dollar in United States. The conversion from local currency to (I$) is made by using the purchasing power parity (PPP) exchange rates. A PPP exchange rate is the number of units of a country’s currency required to buy the same amount of goods and services in the domestic market as the U.S Dollar would by in United States. The PPP exchange rate in 2005 for Bangladesh Taka is 13.03 [9].

### Statistical analysis

Data were recorded and descriptive statistical analysis was done with SPSS version 16.0 system. χ^2^-test was used in the comparison of quantitative data where required. The significance of the results as determined at 95.0% confidence interval (CI) and a value of P < 0.05 was consider to be statistically significant.

### Ethical consideration

The study protocol was approved by the ethical review committee of Dhaka Medical College.

## Results

Out of 3179 patients who visited the epilepsy clinic at DMCH, 854 were selected through the eligibility criteria. The majority of the patients attending outdoor clinic were >11-30 years age group (55.2%) with a mean age of 20.3 ± 9 years. There was a male (53%) predominance (Table 1). Considering the types of epileptic seizure, partial/focal seizure were more common (53%), among whom secondary generalized seizure was the most frequent diagnosis (67% of partial seizure) followed by complex partial seizure (22%). Out of those with generalized epilepsy (44%), generalized tonic clonic seizure was encountered in 74% and absence seizure was observed in 12%. Despite the entire endeavor, about 3% of seizure patients remained unclassified (Table 2).

**Table 1:** Demographic profile of the patients (n = 854)

**Parameter**	**n**	**%**
**Age**		
<10 yrs	167	19.5
11-20 yrs	198	23.2
21-30 yrs	273	32
31-40 yrs	120	14
41-50 yrs	43	5
51-60 yrs	34	4
>60 yrs	19	2.3
**Sex**		
Male	453	53
Female	401	47

**Table 2:** Classification of epileptic seizure (n = 854)

**Parameter**	**n**	**%**
**Generalized epilepsy**	**373**	**44**
GTCS	275	74
Absence	47	13
Tonic	31	8
Atonic	12	3
Myoclonic	8	2
**Partial epilepsy**	**452**	**53**
SPS	50	11
CPS	97	22
Secondary GS	305	67
**Unclassified**	**29**	**3**

GTCS = Generalized Tonic Clonic Seizure, SPS = Simple Partial seizure, CPS = Complex Partial Seizure.

At the end of the follow-up period, the total number of AEDs prescribed were 1144, corresponding to an average of 1.34 AEDs per patient. The number of patients on monotherapy and dual AED therapy were 572 (67%) and 205 (24%) respectively. Polytherapy (i.e. >3 AEDs) was used in only 77 patients (9%). Irrespective of the AED prescription pattern (whether on monotherapy or combination therapy), CBZ (67%) was the most frequently prescribed AED, followed by VPA (43%), PHB (17%), and PHT (8%). About 37% of the patients received CBZ as monotherapy followed by VPA in 21% and PHB in 8% patients. Among the combination therapy, CBZ and VPA was most commonly used (14.5%) as dual therapy and CBZ, VPA and PHT (4%) as polytherapy. Newer generation drugs eg lemotrigine and topiramate was used only as add on therapy in combination with CBZ and VPA (Table 3). Irrespective of the seizure type, the treatment retention rates over the last 2 years for the AEDs in monotherapy varied between 86 and 91% and were highest for CBZ, followed by VPA. Most of the combination regimens had a treatment retention rate of 100%. Effectiveness of the AEDs was also measured as the number of patients being seizure free over this last 2-year period. The effectiveness in terms of reduction of seizure frequency was highest for PHT (100%) and PHB (98%) followed by CBZ (96%) and VPA (95%) (Table 4). Treatment retention rate and seizure freedom rate at the end of follow up across the AED used was statistically significant (*p* value <0.001 for both variables).

**Table 3:** Treatment of epilepsy (n = 854)

**Treatment**	**n**	**%**
**No of AED used**		
Monotherapy	572	67
Two drugs	205	24
Three drugs or more	77	9
**AED Prescribed in clinic**		
CBZ	318	37
VPA	179	21
PHB	68	8
PHT	17	2
**Combination**		
CBZ + VPA	115	13.5
CBZ + PHB	34	4
CBZ + PHT	26	3
VPA + PHB	21	2.5
CBZ + VPA + PHT	34	4
CBZ + PHB + PHT	26	3
CBZ + VPA + TOP	8	1
CBZ + VPA + LTG	8	1

PHB = Phenobarbitone, CBZ = Carbamazepine, VPA = Sodium Valproate, PHT = Phenytoine, TOP = Topiramate, LTG = Lemotrigine.

**Table 4:** Efficacy of AED regimen (n = 854)

**AED regimen**	**Retention rate At the end of F/U (%/ number of patients)**	***P *** **value**	**Seizure free At the end of F/U (%/ Number of patients)**	***P *** **value**
CBZ	91 (287/318)		96 (275/287)	
VPA	86 (154/179)		95 (146/154)	
PHB	22 (15/ 68)		98 (14/15)	
PHT	29 (5/ 17)		100 (5/5)	
CBZ + VPA	100 (115/115)		96 (110/115)	
CBZ + PHB	88 (30/34)	<0.001	90 (27/30)	<0.001
CBZ + PHT	65 (17/26)		76 (13/17)	
VPA + PHB	77 (16/21)		88 (14/16)	
CBZ + VPA + PHT	100 (34/34)		88 (30/34)	
CBZ + PHB + PHT	92 (24/26)		75 (18/24)	
CBZ + VPA + TOP	100 (8/8)		88 (7/8)	
CBZ + VPA + LTG	100 (8/8)		88 (7/8)	

PHB = Phenobarbitone, CBZ = Carbamazepine, VPA = Sodium Valproate, PHT = Phenytoine, TOP = Topiramate, LTG = Lemotrigine.

The average costs of mean daily maintenance dose of AEDs were compared with regard to local currency and International Dollars (I$). PHB and PHT were the cheapest of all AEDs available in Bangladesh. The annual cost of PHB 60 mg dose and PHT 300 mg dose were about 1096 taka and 2190 taka (84 I$ and 168 I$) respectively. The cost of VPA and CBZ was slightly higher (196 I$ and 254 I$). But LTG and TOP were very much expensive (672 I$ and 560 I$) (Table 5). Out of 572 patients on monotherapy, adverse drug reaction (ADR) were observed among 140 (24.5%) of them. The detail of category and type of reaction is given in Table 6. PHT (64%) was the most common drug to cause ADR, especially in the form of gum hypertrophy and CNS manifestations. Though CBZ caused some serious reactions like anaemia, skin rash etc., it was the least common drug to cause ADR (11.6%). About 25.7% patients with VPA and 23.5% patient with PHB experienced ADR. Both of them caused weight gain commonly. Adjustment of drug dose or withdrawal due to ADRs was necessitated in 39% with PHT, 26% with PHB, 9% of patients with VPA and 8% of patients with CBZ in this study.

**Table 5:** Cost of AEDs

**AED**	**Mean maintenance dose (mg/day)**	**Cost per unit mg/tab (Taka**^**1**^**)**	**Annual cost (Taka)**	**Annual cost (International Dollar**^**2**^**)**
CBZ	400	200 (3.5)	2556	196.12
VPA	600	200 (3)	3285	252.06
PHB	60	30 (1.5)	1096	84.10
PHT	300	100 (2)	2190	168.06
TOP	50	25 (10)	7300	560.24
LTG	100	50 (12)	8760	672.28

^1^Local currency.

^2^International dollar is calculated by purchasing power parity (PPP).

**Table 6:** Adverse drug reactions (ADR) with AED monotherapy

		**AED**			
		**N**		**(%)**	
	**Type of reaction**	**CBZ**	**VPA**	**PHB**	**PHT**
		**67 (11.6%)**	**46 (25.7%)**	**16 (23.5%)**	**11 (64%)**
Blood	Aneamia, pancytopenia	15	1	0	0
Skin	Rash, urticaria, photosensitivity, SJS	11	0	0	0
Mouth	Gum hypertrophy/hyperplasia, ulcer	0	0	0	7
GIT	Nausea, vomiting	23	4	7	0
Liver	Hepatitis	14	3	1	0
CNS	Drowsiness, ataxia, vertigo	1	0	0	2
Body weight		17	31	10	9

## Discussion

Epilepsy imposes a huge economic burden on health care system, especially in resource poor countries where the incidence may be as high as 190 per 100,000 people [10]. The Global Campaign Against Epilepsy was established by the International League Against Epilepsy (ILAE), the International Bureau for Epilepsy (IBE), and WHO in order to tackle the management issues related to epilepsy [11]. WHO suggests that medical disorders that require a low technological approach should be managed at the primary health-care level. There are studies from both developed and developing countries on economic analysis of epilepsy which have shown significant economic burden of epilepsy [12]–[14]. These evidences from research in rich countries may or may not accurately predict similar outcome in poor countries. In this context of low priority and high treatment gap of epilepsy care in developing countries, the study was done to establish the low cost and effective first line AED for Bangladesh.

Most of our index population was younger age group (55.2% belonging to 11-30 years age group). The report coincides with Mannan et al. [15] (16-31 years) in Bangladesh. Mac et al. [16] showed a bimodal age distribution of epilepsy patients in developed country with a first peak in childhood and another one in old age. Except for Shanghai in China, most of the Asian countries have younger patients with epilepsy. The probable reason for the missing peak in the older age group in many Asian countries is due to the fact that most of the population are younger compared to number of old people [16,17]. Similar to reports from other Asian countries, there was slightly male predominance [18].

In contrast to the reports from most of the Asian countries, where the IGE ranges from 50-69% and partial seizure from 31-50%, our index population predominantly had partial epilepsy (53%) [19]–[22]. Often it is quite difficult to compare the results of these studies due to lack in application of standardized classification system in epilepsy research in Asia and lack of imaging and electroencephalographic studies, which probably had lead to predominance of IGE in most of the studies. We had the advantage of tertiary care facilities in this context which might be the reason behind dominance of LRE/focal epilepsy in our study. In a similar hospital based study in Srilanka, Senanayake classified 59.7% patients as secondary generalized seizure. Several series of studies from Liberia, Mumbai, Madras, Brazil reported the frequency between 40-50% [23,24].

Monotherapy has been the gold standard of epilepsy treatment for last 20 years. Most (67%) of the patients in our study was successfully maintained on monotherapy. Various first generation AEDs like PHB, PHT, CBZ, VPA and clonazepum are widely used as monotherapy in most of the Asian countries [25]–[30]. A series of trials by Reynolds and Shorvon [31]–[33] also showed similar effectiveness with monotherapy. Not surprisingly CBZ was the most common (67%) AED prescribed as LRE was encountered more frequently. The choice of AED as monotherapy varied between studies, where VPA may be first choice as broad spectrum AED or both used equally [34]–[36]. Conventionally, most of the neurologists prefer VPA for IGE and CBZ for LRE. The Cochrane review [37] provides very little support for this, where VPA showed marginally favorable outcome for IGE. In the Cochrane review of five head-to-head studies in partial epilepsy, CBZ was just superior to VPA. In our study PHT and PHB had the highest level of effectiveness in terms of reduction of seizure frequency, but had very low retention rate than CBZ and VPA due to higher rate of adverse events in the former drugs. The difference was also statistically significant across the AED used in terms of both the treatment retention rate (*p* value <0.001) and seizure freedom rate (*p* value <0.001) at the end of follow up. In contrast to this a published clinical trial from Caucasian and Japanese study showed only 30% retention rate at a median follow up period [38].

PHB was recommended by WHO as the treatment of choice for partial and tonic clonic seizures in resource restricted countries [39]. The strategy had been questioned because of low tolerability with PHB than other antiepileptic drugs [40]. Concerns apply particularly to children, who are especially vulnerable to cognitive and behavioural adverse effects of PHB [41]. But in a study by Banu [42] and colleagues in Bangladesh found no significant difference in behavioral problems such as restlessness and hyperactivity between PHB and CBZ (7% *v* 11%). Though more efficacious in seizure reduction, the treatment retention rate at the end of follow up period on PHB and PHT were very low compared to CBZ and VPA. The discontinuation of former drugs is probably due to their ADR profile. For causality assessment of ADR we only have considered those on monotherapy. Henceforth, we could not evaluate the side effect profile of LTG and TOP, as they were used only as add on therapy. Most adverse effects of AEDs belong to the type A category, that is, they are predictable, dose dependent, and explained by the known pharmacological properties of individual agents [43]. The overall rate of adverse events in our study was higher than that of Mathur et al. [44] (4.67%), but similar to Roopa et al. [45] (10.2%) who reported from a tertiary care hospital like ours. Similar to other studies the ADR was maximum with PHT, followed by VPA and PHB [46]. Most of the side effects correspond well with the known adverse effect profile of PHT [47]. Coinciding our report, Carpay et al. [48] also observed more weight gain with the VPA and PHT, compared o CBZ. VPA has traditionally been associated with a greater incidence of weight gain than that of other AEDs [49]. The percentage of patients requiring changes in their dose regimen due to ADR was comparable for both VPA and CBZ.

This type of cost effectiveness analysis helps in setting the priority for allocation of public health research. Beyond efficacy and cost, availability of AED is also a critical factor for successful implementation of wide range of health care delivery though out the whole nation. The WHO CHOICE [50] work program was under taken in different developing countries to find out effective strategy to combat epilepsy. Such process involved different key parameters like effective treatment coverage, drug price and unit cost of health care service delivery. PHB and PHT are the cheapest AED found in Bangladesh. But the cost of newer generation AEDs like TOP and LTG are six to eight times higher. This is a vital economic factor when the cost of long term treatment is borne mostly by patients themselves.

The major limitations in the study include the retrospective nature and the variability in treatment of epilepsy patients by different neurologists at the epilepsy clinic. We tried to reduce the observer biasness by following the ILAE classification system and common treatment guideline followed by the responsible consultants. We had to exclude a large number of patients who either did not return to follow up or did not complete two years of follow up. The poor follow up may be due to the fact that, the epilepsy clinic of DMCH which is the highest center of referral for patients with epilepsy, encounters patients from all the corners of Bangladesh. Most of them are poor. It often becomes quite difficult for them to come to Dhaka for follow up. Though we have considered the treatment retention rate and number of patient being seizure free at the end of follow up as the measure of effectiveness, the severity of individual seizure events were not considered for analysis. We also could not measure the drug adherence based on medication collection or follow up. Because in Bangladesh there is no database system for recording the medication dispensed or collected by the patients. Moreover, the study from hospital records may not completely represent the scenario in the community.

## Conclusion

The use of older AEDs in primary health care settings is expected to be a very cost effective approach due to lower price and established effectiveness profile. The four conventional old generation drugs (PHB, PHT, CBZ and VPA) recommended by WHO is still effective in our population. Though PHT and PHB are least expensive drugs, their use is limited by the higher rate of adverse event. Henceforth, CBZ and VPA may be effective, less costly and well tolerated drugs in our settings where resources are limited. So the routine use of these four low cost AED may avert the burden of epilepsy treatment in developing and under developed countries.

### Ethics

The study protocol was approved by institutional ethical committee of Dhaka Medical College Hospital.

### Consent

Written informed consent was obtained from the patient for the publication of this report.

## Competing interests

The authors declare that they have no competing interests.

## Authors’ contributions

MH and SUK were involved in planning, consultation, data collection for this study and finally the editing of the manuscript. ATM HH was involved in data analysis and writing the manuscript. The rest were involved in consultation and data collection. All the authors have read and approved the final version of the manuscript.
